# Comprehensive Patient-Specific Intensity-Modulated Radiation Therapy Quality Assurance Comparing Mobius3D/FX to Conventional Methods of Evaluation

**DOI:** 10.7759/cureus.14910

**Published:** 2021-05-08

**Authors:** Amar K Basavatia, Derek A Fiedler, Johannes Ritter, Patrik Brodin, Lee C Goddard, Kyoungkeun Jeong, Shu-Hui Hsu, Dinesh K Mynampati, Ravindra Yaparpalvi, Wolfgang A Tomé

**Affiliations:** 1 Radiation Oncology, Montefiore Medical Center, Bronx, USA; 2 Radiation Oncology, Montefiore Medical Center and Albert Einstein College of Medicine, Bronx, USA

**Keywords:** imrt qa, measurement-based imrt qa, film based imrt qa, log file based imrt qa, imrt, pretreatment quality assurance

## Abstract

Purpose

To determine the appropriateness of implementing Mobius3D/FX (Varian Medical Systems, Inc., Palo Alto, CA, USA) as not only a pretreatment secondary check but as an alternative to measurement-based patient-specific intensity-modulated radiation therapy (IMRT) quality assurance (QA).

Methods

Mobius3D/FX was commissioned and stock beam models were tweaked so that an independent recalculated 3D dose distribution can be obtained. Then, 50 patient-specific treatment plans for various indications were delivered across a 2D ion chamber array, radiochromic film setup, and electronic portal imager and analyzed with MobiusFX and gamma analysis. The concordance of plans scored as passing between MobiusFX and the conventional methods of QA was determined.

Results

All analyzed treatment plans passed with a gamma passing rate >90% across all conventional QA methods, most commonly using a 3%/3mm gamma criterion except for film measurements where a 5%/3mm criterion was applied. There was good agreement and concordance between MobiusFX and conventional methods when using a 3%/3mm criteria for MobiusFX, whereas a 2%/2mm criteria appeared too stringent as it failed treatment plans deemed clinically acceptable using conventional methods.

Conclusions

Using a 50-sample subset of clinically delivered treatment plans this non-inferiority-type comparison shows Mobius3D/FX based on log file analysis to be a suitable alternative to conventional QA methods when utilizing the 3%/3mm gamma criterion. Methods based on log file analysis can provide an opportunity for resource sparing, improving the efficiency, and workflow for evaluating IMRT treatment plans.

## Introduction

It is a standard practice to perform a secondary check of the dose calculation performed by the treatment planning system (TPS) at the completion of every treatment plan in a radiation oncology clinic [1]. This is typically accomplished using a commercial software package but can also be executed in the form of hand calculations. While hand calculations can be adequate for emergency clinical setups and relatively uncomplicated aperture-based static-field treatment plans, they become impractical for complex intensity-modulated radiation therapy (IMRT) and volume-modulated arc therapy (VMAT) treatments which have become standard in most clinics.

Additionally, for every plan that is generated through inverse planning methods, i.e., utilizing an IMRT approach, the expected fluence pattern(s) need to be evaluated. This is currently accomplished in the clinical setting at our institution using an electronic portal imaging device (EPID) if available or using an ion chamber (IC) array. Both methods allow for the recording of a delivered fluence pattern, which can then be comparatively analyzed relative to the expected fluence pattern generated by the TPS. Alternatively, institutions have employed other means of accomplishing these secondary checks using techniques such as radiochromic film [2] measurements or using recorded multileaf collimator (MLC) positions from log files to recreate the delivered fluence pattern [3]. Regardless of the measurement device, all methods typically employ the use of the g-analysis as described by Low et al. [4, 5].

The commercially available Mobius3D/FX (Varian Medical Systems Inc., Palo Alto, CA, USA) system was introduced as an alternative solution to the currently resource-intensive but necessary workflow described above and has been shown to be a useful tool as a secondary check system [6]. The work presented here seeks to determine the appropriateness of implementing Mobius3D/FX not only as a pretreatment, independent, recalculation secondary check but also as a potential alternative to measurement-based patient-specific IMRT quality assurance (QA).

The purpose of this study is to assess the validity of the Mobius3D/FX g-analysis as an alternative method for pretreatment patient-specific IMRT QA. To this end we have performed a non-inferiority study whose positive results show the potential of this system as an alternative to the standard measurement-based IMRT QA workflow, allowing one to streamline the pretreatment clinical workflow with respect to assessing plan safety and deliverability.

## Materials and methods

Commissioning of Mobius3D/FX

The Mobius3D/FX was commissioned and tested as recommended in the physics manual that comes with the system [7]. The Mobius3D/FX system comes with predefined models of each linear accelerator (LINAC) based on the measured percentage depth dose (PDD) for each energy of the specific LINAC. These predefined models are based on MD Anderson’s Imaging and Radiation Oncology Core (IROC) data [8]. This data is the collection, hundreds of LINACs, of annual output measurements and data from phantom measurements from a variety of LINACs across the United States, as part of each center’s accreditation for clinical trial participation. To commission the system for clinical use, one compares the automatically modeled parameters in Mobius3D/FX with the TPS modeled data and output factors. If changes are made in Mobius3D/FX, either for the Dynamic Leaf Gap (DLG) or output factors, then a new beam model has to be computed and compared. As part of the commissioning, there is also a need to check whether the output factors of small MLC fields with large jaw settings match the TPS calculations. Multileaf collimator collimated fields measured with a diode or 0.01cm^3^ IC can be performed for verification of small MLC fields if commonly used. These should agree to at least 90% agreement at 2%/2mm on most fields and at least 90% agreement at 3%/3mm for lesser used fields. Other verification steps include turning off electron contamination and verifying whether depth dose calculations are accurate past dmax.

Patient-specific IMRT QA study design

To determine the non-inferiority of Mobius3D/FX as an alternative method of patient-specific IMRT QA, consistency regarding passing versus failing was tested across a large variety of treatment plans. These methods included radiochromic EBT3 film measurements, EPID-based portal dosimetry, and measurements acquired using an IC array for planar fluence evaluation.

To this end, 50 previously treated external beam treatment plans were analyzed using each of the above-mentioned measurement-based IMRT QA methods. The detailed breakdown of included treatment plans according to the disease site is shown in Table 1. Six treatment sites (prostate, breast, spine stereotactic body radiotherapy (SBRT), head and neck, gynecological, lung) were chosen, with even distribution of treatment plans between four LINACs at our institution. Two LINACs use the M120 MLC, and two LINACs use an HD120 MLC. The g-analyses from these QA methods were subsequently compared to that produced from the log file-based dose recreation using Mobius3D/FX.

**Table 1:** Treatment site and IMRT technique List of all 50 QA plans by anatomical site, type of delivery, number of fields, and the Varian LINAC they were delivered on. Trilogy and Truebeam have HD MLC and both 21EX have Millenium MLC. IMRT: intensity-modulated radiation therapy; SBRT: stereotactic body radiotherapy; VMAT: volume-modulated arc therapy; FiF: field-in-field; LINAC: linear accelerator; HD: high definition; MLC: multileaf collimator

Site	Type	# of Fields	Machine
Prostate	VMAT	2 Arcs	Trilogy
Prostate	VMAT	2 Arcs	Trilogy
Prostate	IMRT	7 Fields	Truebeam
Prostate	VMAT	2 Arcs	Truebeam
Prostate	VMAT	2 Arcs	Truebeam
Prostate	VMAT	2 Arcs	Trilogy
Prostate	VMAT	2 Arcs	Trilogy
Prostate	VMAT	2 Arcs	TrueBeam
Prostate	IMRT	7 Fields	21-EX
Prostate	IMRT	7 Fields	21-EX
Breast	IMRT	4 Fields	Trilogy
Breast	IMRT	4 Fields	21-EX
Breast	IMRT	2 Fields	Trilogy
Breast	IMRT	5 Fields	21-EX
Breast	FiF	2 Fields	21-EX
Breast	IMRT	4 Fields	Truebeam
Breast	FiF	2 Fields	Truebeam
Breast	IMRT	2 Fields	21-EX
Breast	FiF	2 Fields	21-EX
Breast	FiF	2 Fields	Trilogy
Head and Neck	IMRT	5 Fields	21-EX
Head and Neck	VMAT	3 Arcs	Truebeam
Head and Neck	IMRT	5 Fields	Trilogy
Head and Neck	IMRT	9 Fields	Trilogy
Head and Neck	IMRT	9 Fields	Truebeam
Head and Neck	IMRT	9 Fields	21-EX
Head and Neck	IMRT	6 Fields	21-EX
Head and Neck	IMRT	9 Fields	21-EX
Head and Neck	VMAT	2 Arcs	Truebeam
Head and Neck	IMRT	9 Fields	Trilogy
Gynecological	IMRT	12 Fields	21-EX
Gynecological	VMAT	2 Arcs	21-EX
Gynecological	IMRT	7 Fields	Trilogy
Gynecological	IMRT	7 Fields	Trilogy
Gynecological	IMRT	7 Fields	21-EX
Gynecological	IMRT	7 Fields	Truebeam
Gynecological	IMRT	7 Fields	Truebeam
Gynecological	IMRT	9 Fields	21-EX
Gynecological	IMRT	7 Fields	21-EX
Gynecological	VMAT	2 Arcs	Trilogy
Spine SBRT	VMAT	2 Arcs	Truebeam
Spine SBRT	VMAT	2 Arcs	Truebeam
Spine SBRT	VMAT	2 Arcs	Trilogy
Spine SBRT	VMAT	2 Arcs	Trilogy
Spine SBRT	VMAT	2 Arcs	Truebeam
Lung SBRT	VMAT	2 Arcs	Trilogy
Lung SBRT	VMAT	2 Arcs	Trilogy
Lung SBRT	VMAT	2 Arcs	Trilogy
Lung SBRT	VMAT	2 Arcs	Truebeam
Lung SBRT	VMAT	2 Arcs	Truebeam

Mobius3D and MobiusFX QA analyses

Utilizing a proprietary collapsed cone convolution/superposition algorithm [8], the Mobius3D system performed independent dose calculations and recalculations for 50 patient treatments exported from Eclipse v.11 with AAA computations (Varian Medical Systems Inc., Palo Alto, CA, USA). At the end of each plan check, results for each plan are compared to the Eclipse-generated dose distribution. Target coverage and dose constraints for organs at risk are checked, and a three-dimensional gamma comparison of TPS to Mobius3D dose agreement has been performed. 

To limit the gamma comparison with respect to clinically relevant voxels, any voxel with a density value of less than 0.2g/cm^3^ is not included unless it is within a contoured structure with the word “lung” in its name [7]. Similar to gamma calculations for single plane analysis used in the other portions of this work, anything below 10% of the maximum dose measured was excluded from the analysis. 

**Figure 1:**
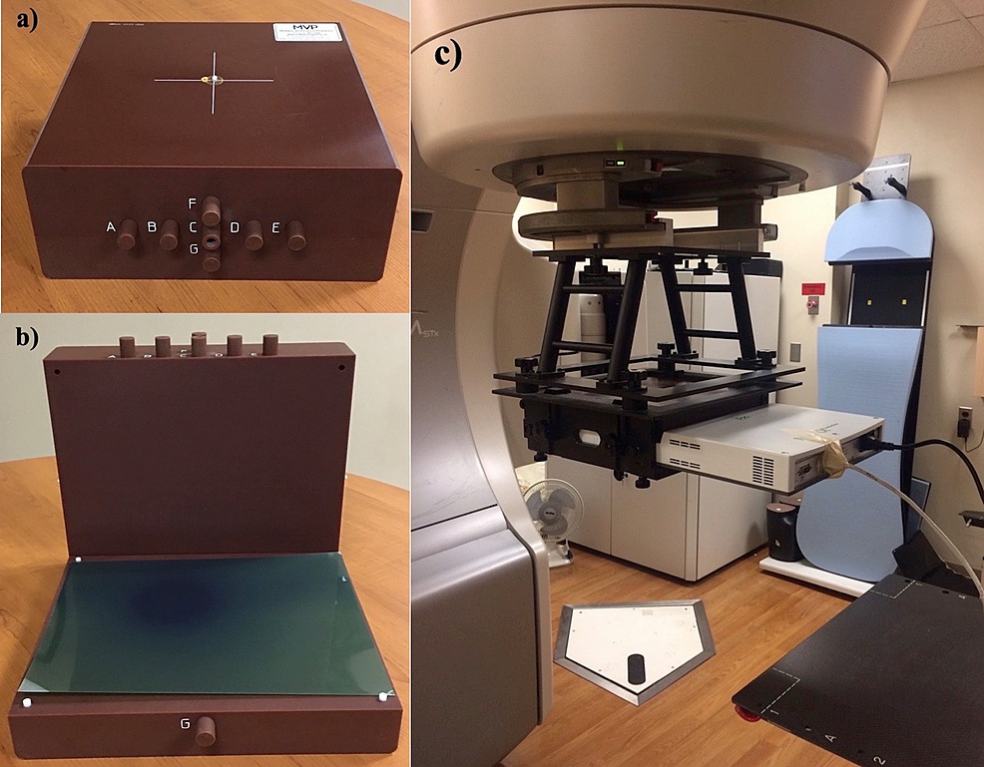
Mobius Verification Phantom and MatriXX Ion Chamber Array Illustration of the measurement setup for a) the Mobius Verification Phantom (MVP) with chamber inserts b) Gafchromic film placement within the MVP and c) Matrixx ion chamber array with gantry holder and build-up.

After each of the patient-specific QA measurements performed as described below, MobiusFX was run in parallel analyzing each of the delivered treatment plans for comparison. This was done by automatically acquiring the written log files and performing a dose calculation or recalculation using the recorded machine parameters. The MobiusFX analysis also includes a section in the initial fraction check, which details the expected results if the delivery was instead delivered to the Mobius Verification Phantom (MVP) (Figure 1a). The MVP was used in this study for IC and EBT3 film measurements and is discussed later.

Ion chamber array measurements

The IBA Dosimetry (Schwarzenbruck, Germany) MatriXX-Evolution was used for all IC array measurements. Consistent with other array detectors, the MatriXX device is calibrated to collect and record a fluence pattern of the delivered plan with which to compare to a digitally created fluence pattern within a clinic’s respective TPS. The MatriXX array contains 1020 ionization chambers in a 24x24 cm^2^ active area, the relative spatial resolution is considered high for an array type verification tool. 

For the purposes of this study, each verification plan was generated in Eclipse using the built-in treatment verification module. Our clinic uses a standard setup for every machine with the array set to 100cm SSD, and then a 5cm solid water slab placed on top of the active volume. Each LINAC has its own unique MatriXX calibration, performed by delivering a 10x10 cm^2^ photon field using the described measurement setup delivering 100 monitor units. Before any treatment verification plans were delivered, a standard measurement was made and compared to the expected calibration value to ensure appropriate QA measurements were obtained.

Verification plans were delivered and analyzed as a composite beam output, i.e., not per field. The MatriXX was mounted on the gantry mount (Figure 1c), and thus the device and beam were perpendicular at each gantry angle, thus a perpendicular composite was obtained. After all data were collected, the exported fluence plane from the TPS was compared with the measured dose data. The imported plane from the TPS is normalized, relative to the maximum value collected by the MatriXX and utilizes a 10% of maximum value cut-off level for the gamma calculation, excluding points below this threshold. Each comparison was performed using a 3%/3mm gamma criteria. 

EPID-based portal imaging

Varian Medical Systems standard production EPIDs were utilized for portal imager-based IMRT QA acquisitions. Of the four linear accelerators used in this work, one utilizes an aS500 model (resolution of 512 $\times$ 384 pixels) and three utilizing an aS1000 model (resolution of 1024 $\times$ 768 pixels), both characterized by 16-bit grayscale images. 

Each patient-specific IMRT QA plan was generated in Eclipse v11 using the built-in treatment verification feature. All measurements were taken with the treatment couch positioned out of the field and were collected as individual beam acquisitions in a perpendicular field-by-field arrangement. Each beam is therefore analyzed separately using theARIA Portal Dosimetry software application (Varian Medical Systems, Inc., Palo Alto, CA, USA). Each comparison was performed using a 3%/3mm gamma criteria. 

Radiochromic EBT3 film

Film-based IMRT QA is a well-studied and understood method of treatment fluence verification, albeit resource demanding [9, 10]. While the symmetrical structure of the film used removes the necessity of consistency in scanner placement orientation, for the purposes of this work orientation was kept constant for all film scans.

In this study, Gafchromic EBT3 (Radiation Products Design, Albertville, MN, USA) (batch numbers 03311401 and 09071603) was used for all irradiation procedures. Films were handled and calibrated according to a published protocol [11].

Films were exposed using the MVP as a structure for supplying both a standard setup and providing appropriate buildup (Figure 1b). Treatment fields were delivered using all parameters used for the treatment of a patient, i.e., gantry, collimator, and table rotation, so that a true composite was obtained. After delivery of the treatment fields, the Mobius software automatically acquired the log files. Mobius3D/FX then recreates or recalculates the dose distribution per the generated fluence pattern expected from the log file data. At the completion of MobiusFX’s automated analysis of this first delivered fraction, a “Phantom Verification” section is accessible. The expected doses at the various chamber slots in the MVP are presented as previously described in the section on 'Patient-specific IMRT QA study design', as well as a film plane which can be downloaded as a DICOM (Digital Imaging and Communications in Medicine) file and compared to the acquired film measurement. It is this generated film plane that was used for all film-based IMRT QA comparative analyses. 

Statistical analysis

Results of the gamma analyses performed either using the three measurement-based conventional QA methods or using MobiusFX are presented as the percentage of failing points at the specified gamma criteria, e.g., 3%/3mm. The non-inferiority of using MobiusFX as compared to conventional QA methods was assessed by determining the agreement between plans scored as passing/failing at the >90% gamma pass rate, and the concordance of plans scored as passing between MobiusFX and the conventional methods. Although there is no formal threshold that would infer non-inferiority in this setting, good agreement and a high concordance for passing plans would suggest that using Mobius FX performs similarly compared to the other QA methods. While TG-218 recommends a 95% gamma pass rate with 3%/2mm parameters, TG-119 on the other hand recommends a 90% gamma pass rate with 3%/3mm parameters [12, 13]. All analyses were performed using MATLAB v.2018a (The MathWorks, Inc., Natick, MA, USA)

## Results

**Figure 2:**
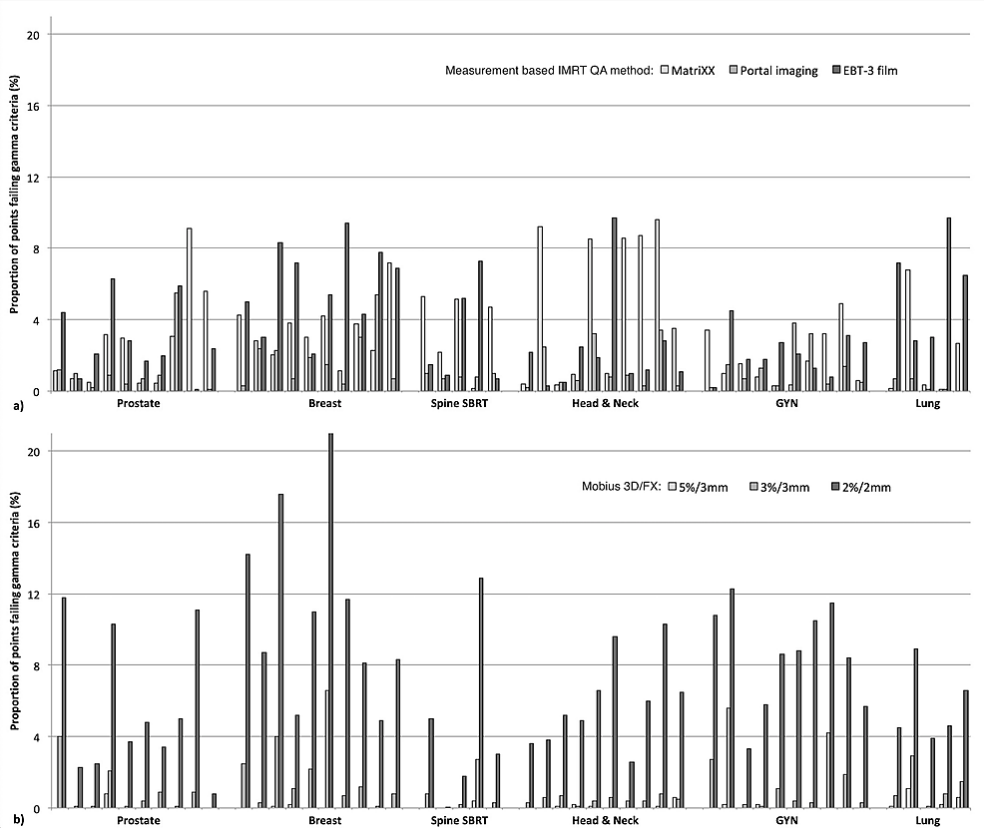
QA Results a) Proportion of points failing the three measurement-based IMRT QA methods using a 3%/3mm gamma criteria except for EBT-3 film measurements where a 5%/3mm criteria was applied. b) Proportion of points failing using gamma criteria of 5%/3mm; 3%/3mm; and 2%/2mm for the MobiusFX system. IMRT: intensity-modulated radiation therapy; SBRT: stereotactic body radiotherapy; GYN: gynecological; QA: quality assurance

We found that all analyzed treatment plans passed the >90% gamma passing rate across all conventional QA methods, most commonly using a 3%/3mm gamma criterion, except for EBT-3 film measurements where a 5%/3mm criterion was applied. The QA results from the conventional methods are presented in Figure 2a as the percentage of points failing the gamma analysis, with EPID-based portal imaging showing the lowest amount of failing points in general. The QA results for breast and head and neck plans tended to have the highest amount of failing points when comparing plans across different anatomical sites. Similarly, the QA results using various gamma criteria for the MobiusFX system are presented in Figure 2b. Here, it is clear that a 2%/2mm gamma criterion is too stringent as it would reject a number of treatment plans (based on a minimum of 90% passing points) that were shown to pass conventional QA methods. 

Determination of the most relevant gamma criteria when evaluating the MobiusFX system in this non-inferiority setting was based on the results presented in Table 2. It is clear that there is good agreement and concordance of passing plans when using 90% of points passing criteria for the 5%/3mm and 3%/3mm levels. However, we are showing in this table a more stringent >95% of points passing criteria for the 5%/3mm and 3%/3mm levels. The 2%/2mm criterion again appears too stringent, and when combined with the results from Figure 2b, the 5%/3mm criterion is likely not a good criterion either as it does not find any failing points, and might not catch delivery errors that are identified using conventional QA methods. This inability to catch inconsistencies between the planned and delivered dose expectation would render MobiusFX an inferior QA system if a 5%/3mm criterion is used. Conversely, the 3%/3mm criterion shows good agreement with conventional QA methods and also provides more relevant plan-specific results, and thus appears to be a good, non-inferior alternative to conventional QA.

**Table 2:** Measures of Agreement Between Plans Measures of agreement between plans scored as passing by either of the three measurement-based QA methods compared to varying levels of gamma criteria when analyzed using Mobius. Passing is defined as >95% of points passing the gamma criteria, while concordance of passing QA plans is defined as the percentage of plans passing the indicated measurement-based method at the indicated level that also pass Mobius 3D/FX at the indicated. EPID: electronic portal imaging device; QA: quality assurance

Any of the 3 measurement measurement-based methods	Mobius (5%/3mm)	Mobius (3%/3mm)	Mobius (2%/2mm)
# of plans passing any measurement-based QA at the given threshold: 50/50	# plans passing: 50/50	# plans passing: 48/50	# plans passing: 20/50
Agreement, n (%)	50 (100%)	48 (96%)	20 (40%)
Concordance of passing QA plans (%)	100%	96%	40%
EPID (3%/3mm)			
# of plans passing this QA method at the given threshold: 48/50	# plans passing: 50/50	# plans passing: 48/50	# plans passing: 20/50
Agreement, n (%)	48 (96%)	46 (92%)	18 (36%)
Concordance of passing QA plans (%)	100%	96%	38%
MatriXX (3%/3mm)			
# of plans passing this QA method at the given threshold: 39/50	# plans passing: 50/50	# plans passing: 48/50	# plans passing: 20/50
Agreement, n (%)	39 (78%)	37 (74%)	21 (42%)
Concordance of passing QA plans (%)	100%	95%	38%
Gafchromic Film (5%/3mm)			
# of plans passing this QA method at the given threshold: 36/50	# plans passing: 50/50	# plans passing: 48/50	# plans passing: 20/50
Agreement, n (%)	36 (72%)	36 (72%)	24 (48%)
Concordance of passing QA plans (%)	100%	97%	42%

## Discussion

In this non-inferiority study employing both Varian’s C-Series and TrueBeam/Edge accelerators, the Mobius system has been shown to be a clinically relevant tool when utilizing the 3%/3mm gamma evaluation. The end goal is the same as with conventional QA and that is to evaluate whether a treatment plan is clinically deliverable. Each of the methods utilized to test that treatment plan has a different QA setup and an apples-to-apples comparison is not possible. However, the trend for the magnitude of failures and the overall pass/fail results are in good agreement in our analysis. Thus, with non-inferiority, the goal of showing whether the “experimental” system, i.e., Mobius, is not a worse tool for patient-specific IMRT QA compared to conventional methods was achieved. In addition, in a recent study, the Mobius system was examined for sensitivity and specificity and was shown to be more sensitive than the typical IMRT QA methods [14]. Sensitivity is referring to the proportion of unacceptable plans (i.e., the proportion that failed or had poor results).

When comparing the gamma passing results of each respective patient-specific plan evaluation method, it is important to consider and evaluate the specific differences of each modality. Mobius utilizes a three-dimensional gamma check of calculated voxel dose, determined from dynalog and delivery files written at the time of beam delivery at the LINAC. The conventional QA methods employed here are all planar based (two dimensional), collecting a fluence pattern at a known plane, and then comparing that pattern to the expected.

Each of the three planar check methods -- MatriXX, EPID based, and film -- are comparing their respective collection planes at different positions and in different media. A film check requires a chemical reaction initiated by ionizing radiation to produce a distribution of optical densities, which is converted to dose through a user-generated calibration curve. The MatriXX is an array of ion chambers with its own set of correction factors and resolution limitations per the type and number of measurement sites. An EPID dosimetry-based measurement, similar in data density to film, determines an absolute measurement termed a calibration unit with its own uncertainty. 

While each planar check method has its own sources of uncertainty, it, however, benefits from directly measuring the delivery output. Mobius checks log files written during beam delivery, reproducing expected delivery indirectly ascertaining what has occurred through calculation. Therefore, no inconsistencies regarding beam output can be directly observed using Mobius, and a rigorous machine QA program must be established to ensure that the information in the log files is sufficiently accurate. Implementing TG-142-based calibration criterion for at least the jaws and MLC positions is necessary, including tests such as confirming jaw position to be within 1mm accuracy and performing weekly picket fence tests which ensure leaves are within ±0.3mm of expected positions. 

To put our study in the context of previous work, log file analyses have been performed and compared with PTW Octavius [15], SunNuclear Mapcheck, ArcCHECK and 3DVH, IBA Compass, and the Scandidos Delta4. A number of investigators have employed in-house log file analyses, fluence, and dose reconstruction using MATLAB [16, 17]. For instance, Saito et al. [16] have employed an in-house developed dynalog based dose reconstruction system and have compared it to ArcCheck-3DVH for different anatomical sites establishing good agreement utilizing gamma criteria ranging from 3%/3mm to 1%/2mm. Sun and colleagues [17] have evaluated 16 IMRT plans to validate MLC delivery performance using Mapcheck and a water phantom with IC and compared to log files analyzed using MATLAB showing good agreement at the 2% and 3% action levels. While Defoor and colleagues [18, 19] employed in-house log file analysis to compare it to EPID and Delta4 for a set of 15 VMAT patient plans. Moreover, Nelson and colleagues [20] compared 12 IMRT and VMAT plans delivered on an Elekta accelerator. These deliveries were then analyzed using Mobius3D/FX and compared to those obtained using Mapcheck. They found an average pass rate of 97.1% at the 3%/3mm level using Mapcheck and a pass rate of 96.9% using the same criteria for Mobius3D/FX. As is the case for our study, these studies clearly show the utility of log file-based QA for IMRT treatment plan verification. Our study, looking at a variety of anatomical sites and comparing log file-based analysis to detector array-based IMRT QA, film, and EPID further strengthens the findings of these investigators by showing that log file-based IMRT QA is a viable option for pretreatment IMRT QA.

Similarly, Clemente-Gutierrez et al. [21] have tested Mobius3D against the IBA Compass system utilizing 12 VMAT plans as well as analyzing TG 119 test cases showing that the two systems performed similarly. These findings were confirmed by Fontenot et al. [22] utilizing TG-119 dosimetric goals and looking at VMAT and IMRT plans with IC measurements showing good agreement with Mobius3D/FX. Furthermore, our results corroborate the findings of Rangaraj et al. [23], who have compared in-house developed log file QA tools to widely adopted 2D planar dosimetry QA methods showing similar results to ours. Therefore, while some argue that phantom-based IMRT QA measurements should continue to be made [24], our work and the work discussed above indicate that log file-based QA methods present a suitable alternative.

While we have employed in this work the gamma index tool as the evaluation tool across all IMRT QA methods as the basis for comparison amongst the different QA methods, it should be noted that the gamma index tool is not without its limitations [25]. However, Mobius3D/FX also allows dose‐volume histogram (DVH)-based metrics for independent plan evaluation and per fraction DVH metric evaluation, which could provide some further utility for patient-specific plan evaluation. However, DVH metrics could not be evaluated with all the above-mentioned conventional QA methods and compared directly, thus the gamma index tool was utilized. Moreover, it should be mentioned that neither Tomotherapy, CyberKnife, MR LINACs, nor Gamma Knife were part of this study and hence lie outside the scope of this analysis.

## Conclusions

Using a 50-sample subset of clinically delivered plans our non-inferiority type comparison shows Mobius3D/FX to be a suitable alternative to conventional QA methods when utilizing the 3%/3mm gamma evaluation as the universal tolerance limit for one’s clinic. The effectiveness of log file analysis shows that patient specific quality assurance of intensity modulated radiation therapy plans can safely be evaluated. A carefully constructed and commissioned quality assurance program should have multiple means of detection and overlap for safety. Once in place, it should be safe to use log file analysis for IMRT QA for LINACs.
